# Index of oral health literacy as the instrument for development of personalized prevention programs of stomatologic diseases

**DOI:** 10.1186/1878-5085-5-S1-A120

**Published:** 2014-02-11

**Authors:** Vasily Alyamovskiy, Anatoly Duzh, Olga Sokolova

**Affiliations:** 1 Krasnoyarsk State Medical University named after Prof. V.F. Voino-Yasenetsky, Russian Federation

## 

According to World Health Organization (WHO), dental caries is common among 60-90% of school children and nearly among 100% of adults. Further, severe form of periodontal disease is found in 15-20% of 35-44 years old patients. WHO experts emphasize that timely prevention of dental diseases will not only contribute importantly to mainitaining oral health but would also significantly reduce cost of care and treatment of oral diseases.

An important component in the prevention of oral diseases is informing of patients about the possibility of maintaining oral health with the help of available oral hygiene products. For adequate and efficient planning of activities to raise oral health awareness it is necessary to estimate level of oral health literacy of the community. In different countries there are certain questionnaires which are primarily used during scientific research. Many of these questionnaires and tests are not well-suited for use in patients with different culture and language.

We suggest that standardization of oral health literacy assessment would be conducive to more effective evaluation of oral health care needs of a given population. Thus, we have developed an index of oral health literacy which has the following characteristics:

- questions are universal and reflect current trends of prevention of oral diseases;

- questions have no cultural contexts and attachments;

- questions can be translated into any language;

- questions have some variants for answers;

- quantitative assessment of oral health literacy.

Pilot studies showed that the developed index of oral health literacy can reliably predict the level of knowledge in young patients and the state of the oral health.

In conclusion, the use of the developed index should will allow assessment of oral health literacy of patients regarding aspects of oral health preservation irrespective of nationality and language should facilitate the development of personalized approaches to prevention of dental diseases.

